# Highly variable cancer subpopulations that exhibit enhanced transcriptome variability and metastatic fitness

**DOI:** 10.1038/ncomms11246

**Published:** 2016-05-03

**Authors:** Alexander Nguyen, Mitsukuni Yoshida, Hani Goodarzi, Sohail F. Tavazoie

**Affiliations:** 1 Laboratory of Systems Cancer Biology, The Rockefeller University, 1230 York Avenue, New York 10065, USA

## Abstract

Individual cells within a tumour can exhibit distinct genetic and molecular features. The impact of such diversification on metastatic potential is unknown. Here we identify clonal human breast cancer subpopulations that display different levels of morphological and molecular diversity. Highly variable subpopulations are more proficient at metastatic colonization and chemotherapeutic survival. Through single-cell RNA-sequencing, inter-cell transcript expression variability is identified as a defining feature of the highly variable subpopulations that leads to protein-level variation. Furthermore, we identify high variability in the spliceosomal machinery gene set. Engineered variable expression of the spliceosomal gene *SNRNP40* promotes metastasis, attributable to cells with low expression. Clinically, low SNRNP40 expression is associated with metastatic relapse. Our findings reveal transcriptomic variability generation as a mechanism by which cancer subpopulations can diversify gene expression states, which may allow for enhanced fitness under changing environmental pressures encountered during cancer progression.

## Abstract (web summary)

Phenotypic and genetic intra-tumor heterogeneity have an important role in cancer progression and therapeutic resistance. Here, the authors show that phenotypically variable tumor subpopulations exhibit higher metastatic potential and display enhanced intra-clonal transcriptomic variability, likely promoted by deregulated spliceosome activity.

## Graphical abstract

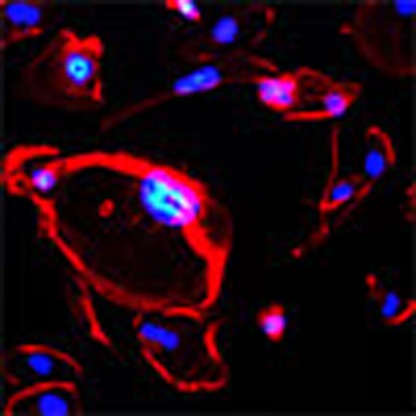

A given cancer type can display tremendous variation from patient to patient, while within a patient, individual neoplastic lesions often grow at different rates and respond differentially to the same therapy. Even within a given tumour, individual cells can display substantial variation at the genetic^1^,^2^,^3^, epigenetic^4^,^5^ and phenotypic levels^6^,^7^. This heterogeneity might be particularly beneficial when cancers face strong selective pressures such as chemotherapy^8^,^9^ or metastatic barriers^10^,^11^,^12^. Notably, functional variability can be sustained over time without genetic changes^8^, suggesting epigenetic control or other mechanisms as paths to molecular variability generation^13^. Many important studies on tumour heterogeneity have provided static snapshots of genetic heterogeneity^1^,^2^; however, functional and phenotypic characterization of individual clones within a tumour population can provide insights into the molecular and cellular features that propagate heterogeneity and diversity generating capacity^14^.

Despite its pervasiveness in cancer, the mechanisms, aside from genetic mutations, that mediate phenotypic heterogeneity generation in driving cancer progression remain poorly understood. These mechanisms may contribute to the evolution of cancer populations, leading to heritable variation that provides fitness advantages under varying selective pressures^15^. Furthermore, it is not known whether phenotypic diversity among cancer cells within a population is molecularly regulated or whether it is simply an epi-phenomenon^16^. To generate an experimental model wherein genetic variation between cells is minimized so that non-genetic contributions to heterogeneity generation can be assessed, we have derived isogenic, clonal subpopulations from human cancer populations. Here we have discovered clonal subpopulations of cells that display high morphological variation. These subpopulations displayed variability of multiple phenotypes, and this feature was inherited by their single cell progeny. Highly variable (HV) subpopulations exhibited increased metastatic capacity and survival in the presence of chemotherapies, consistent with diversification-enabling enhanced fitness. Furthermore, in human breast cancers, nuclear morphological variation was found to associate with clinical metastasis. Molecular analyses revealed that highly variable subpopulations exhibit genetic stability, yet express enhanced cell-to-cell transcriptomic variability, which is transmitted to the protein level. Finally, gene set enrichment analysis revealed spliceosomal machinery components to display high-transcript expression variability, suggesting a means by which variation could be transmitted to a global level. Spliceosomal gene set expression variability is consistent with the increased pre-mRNA variability observed in these subpopulations. Indeed, engineered variation of the SNRNP40 spliceosomal gene's expression among cells within a breast cancer population promoted their metastatic fitness. Further analysis revealed cell populations with low SNRNP40 expression exhibit enhanced metastatic capacity, displayed gene expression changes consistent with that seen in highly variable subpopulations, and contained increased unspliced pre-mRNAs. Clinically, low SNRNP40 expression was found to be associated with metastatic outcomes. These findings highlight an aspect of intra-clonal tumour heterogeneity that has not yet been previously addressed. The experimental model established here can be applied to various cancers to better understand non-genetic contributions to heterogeneity and to study the impact of such deregulation among cancer populations and their progeny.

## Results

### Isolation of clonal subpopulations with morphologic variation

**Figure 1:**
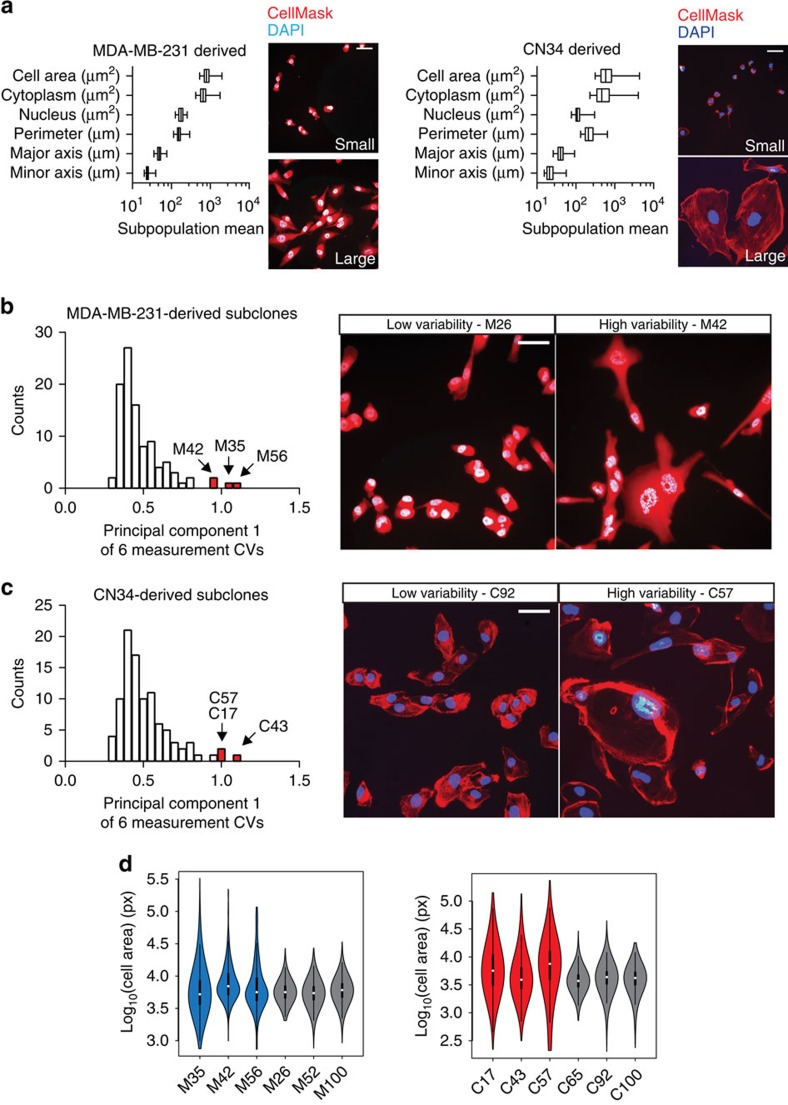
Clonal subpopulations generate morphological diversity. (**a**) Clonal subpopulations were generated, labelled with CellMask stain to label entire cells and DAPI dye to label nuclei, imaged, and analysed for six size parameters using CellProfiler software. Summarized size parameters are shown from MDA-MB-231 (left, *n*=98) and CN34 (right, *n*=97) breast cancer cell lines. Median, interquartile range, minimum and maximum are depicted by box plots. Representative images of small-sized and large-sized cell populations are shown; scale bars are 50 μm. (**b**,**c**) Histograms (left) of the first principal component of size coefficient of variation (CV) was used to assess subpopulation size heterogeneity in MDA-MB-231-derived clones (**b**) and CN34-derived clones (**c**). Candidate high (red) variability populations are indicated. Representative images of high and low variability populations are shown (right); scale bars are 50 μm. (**d**) Distribution of cell area from candidate high and low variability populations in MDA-MB-231-derived clones (left) and CN34-derived clones (right). Median, interquartile range, minimum and maximum are depicted by violin plots.

To study phenotypic diversity in cancer cells, we derived nearly 200 clonal subpopulations from 2 breast cancer cell lines and assessed these subpopulations for intra-clonal heterogeneity in cell size through automated image analysis of 29,390 cells in total using CellMask stain to label entire cells and DAPI dye to label nuclei. Subpopulations, derived from the human cancer cell line MDA-MB-231 (MDA) and the minimally passaged primary CN34 breast cancer line (CN), displayed inter-clonal variation in six size parameters (Fig. 1a). To quantitatively assess intra-clonal size heterogeneity, coefficient of variation for each subpopulation was calculated for each size parameter, and principal component analysis was performed for each parental line. The majority of clonal subpopulations displayed a range of variability as assessed by using the first principal component—consistent with a single peak distribution (Fig. 1b,c). A few subpopulations demonstrated exceptionally high intra-clonal, cell-to-cell size variation without exhibiting significant differences in their population-level means (Fig. 1b–d).

### Highly variable subpopulations exhibit phenotypic diversity

**Figure 2:**
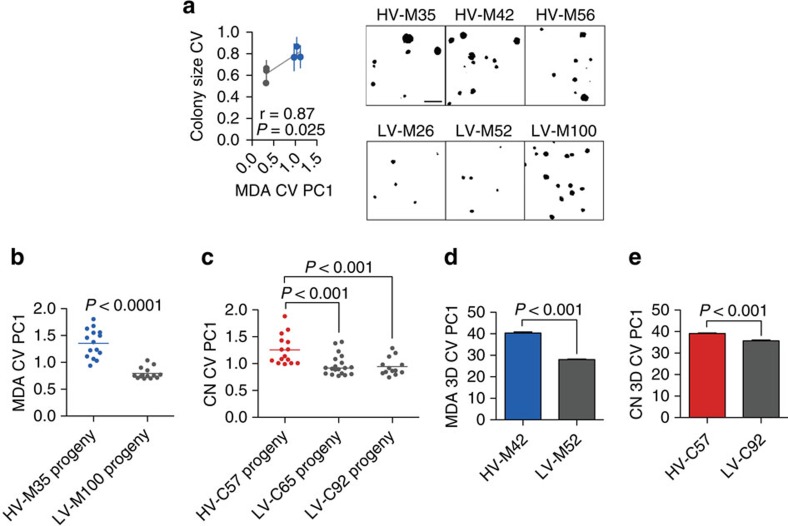
Phenotypic characterization of highly variable subpopulations. (**a**) MDA-derived colonies were grown in triplicate plates and measured for variability in colony area. Error bars indicate s.e.m. of three independent experiments. *P* values were generated by testing Pearson's correlation coefficient with two-sides. Representative images of colonies stained by with crystal violet and thresholded in ImageJ are shown on right; scale bar, 5 mm. (**b**,**c**) Single cells isolated from indicated MDA-MB-231 (**b**) and CN34 (**c**) subpopulations were expanded into clonal populations and were assessed for cell size heterogeneity. Lines represent median. *P* values were derived using two-sided Mann Whitney *U* test. (**d**,**e**) The first principal component of 3D size coefficient of variation was used to assess 3D size heterogeneity from indicated MDA-MB-231 (**d**) and CN34 (**e**) subpopulations. Error bars indicate s.e.m. of three independent experiments. *P*-values were derived using two-sided *t*-test.

To determine whether these highly variable subpopulations could give rise to and maintain phenotypic diversity beyond cell size, we assessed proliferative variability as an independent functional measure by colony formation assays. We first confirmed that cell size and cell density within colonies were not contributing factors to colony area differences (Supplementary Fig. 1b). While bulk population growth in culture was similar between subpopulations (Supplementary Fig. 1a), MDA-derived subpopulations that exhibited high intra-clonal size variation also displayed high variability in proliferative capacity, which could be visualized as high variation in colony sizes (Fig. 2a). Among the CN-derived populations, only subpopulation C57 displayed a modest increase in proliferative variability (Supplementary Fig. 1c,d), suggesting that the other morphologically heterogeneous subpopulations from this parental line may not exhibit variation of additional phenotypes beyond morphology. These subpopulations, herein referred to as highly variable (HV) subpopulations, maintained high size variability in progeny clonal subpopulations when compared with lowly variable (LV) subpopulations (Fig. 2b,c). These HV subpopulations maintained enhanced size variability when measured in three dimensions (Fig. 2d,e), after passage in culture (Supplementary Fig. 2a), and after seeding at various densities (Supplementary Fig. 2b) with no consistent difference in cell cycle phasing (Supplementary Fig. 2c). In addition, to determine whether HV cells over-express markers associated with what some propose to be the ‘cancer stem cell state', we assessed the expression levels of breast cancer stem cell markers CD44 and CD24 by flow cytometry and observed no difference between HV and LV subpopulations for these markers (Supplementary Fig. 2d–f). All in sum, these clonal subpopulations displayed an enhanced and heritable ability to generate diversity across multiple phenotypic dimensions.

### Highly variable subpopulations exhibit metastatic fitness

**Figure 3:**
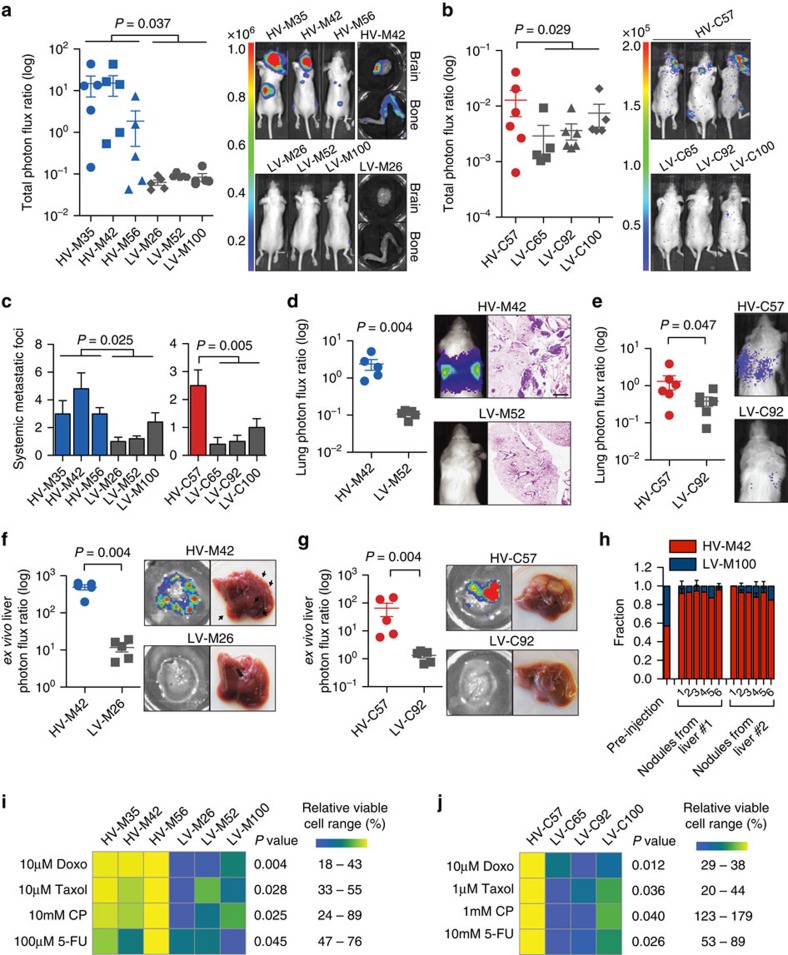
Highly variable subpopulations display enhanced metastatic capacity and survival in the presence of various chemotherapies. (**a**,**b**) Bioluminescence quantification of total body metastatic burden by 4 × 10^4^ MDA-derived cell populations after 42 days (*n*=5) (**a**) and 2 × 10^5^ CN-derived cell populations after 72 days (*n*=6) (**b**) following inoculation into arterial circulation via intracardiac injection. *P*-values were derived using two-sample (MDA, left) and one-sample (CN, right) one-sided *t*-test. Representative mouse and organ bioluminescence are shown. (**c**) Systemic metastatic foci per mice were counted as distinct, minimal bioluminescence signals. *P* values were derived using two-sample (MDA-derived) and one-sample (CN-derived) one-sided *t*-test. (**d**,**e**) Bioluminescence quantification of lung metastases by 4 × 10^4^ MDA-derived cell populations after 70 days (**d**) and 2 × 10^5^ CN-derived cell populations after 112 days (**e**) following inoculation into the tail vein. *P* values were derived using one-sided Mann Whitney *U* test; *n*=5. Representative bioluminescence and lung histology is shown. Scale bar, 1 mm. (**f**,**g**) *Ex vivo* bioluminescence quantification of liver metastases by 4 × 10^4^ MDA-derived cell populations after 39 days (**d**) and 2 × 10^5^ CN-derived cell populations after 91 days (**g**) following inoculation into the portal circulation. *P*-values were derived using one-sided Mann–Whitney *U*-test; *n*=5. Representative bioluminescence and gross histology are shown. (**h**), 5 × 10^5^ cells consisting of equal parts of HV-M42 cells, labelled with blasticidin resistance gene, and LV-M100 cells, labelled with puromycin resistance gene, were inoculated into the portal circulation. Liver metastases were extracted after 28 days and processed for qPCR quantitation of genomic DNA resistance genes. Error bars indicate s.d. (**i**,**j**) 5 × 10^3^ MDA-derived (**i**) and CN-derived (**j**) subpopulations were each seeded in triplicate and treated with drugs 24 h after seeding. After 48 h, WST-1 reagent (Roche) was added to assay viable cells. Heat map scaling is for each compound and is shown on the right. *P*-values were derived using two-sample (**i**) or one-sample (**j**) one-tailed *t*-test. Doxo, doxorubicin; Taxol, paclitaxel; CP, cyclophosphamide; 5-FU, 5-fluorouracil. Error bars indicate s.e.m. unless otherwise indicated.

Metastatic colonization of an end organ represents a major bottleneck during cancer evolution that would greatly benefit from diversity generation and is clinically responsible for the majority of cancer deaths^17^,^18^. Our identification of subpopulations of cells with high versus low diversification potential from the same individual patients' cancer populations allowed us to directly test the impact of intra-clonal diversification ability on metastatic colonization capacity. HV subpopulations exhibited enhanced systemic metastatic capacity when introduced into the arterial circulation of mice as determined by total tumour burden (Fig. 3a,b). In addition, this increased tumour burden was in large part attributed to an increased number of systemic metastatic foci (Fig. 3c), indicating an increased frequency of colonization of systemic sites as opposed to an increased growth rate of formed metastases. Moreover, HV subpopulations derived from both human lines more efficiently colonized the lung on venous inoculation (Fig. 3d,e) and the liver on portal circulation injection (Fig. 3f,g), indicating that the enhanced metastatic colonization capacity is broadly applicable to multiple organs posing diverse selective barriers. In addition, portal circulation inoculation of a mixed population consisting of an equal number of HV and LV cells revealed HV cells to contribute between 81.2 and 99.6% of formed liver metastases (Fig. 3h), demonstrating that HV cells maintain an increased metastatic capacity in a mixed population. Because broad diversity generation has the potential to enhance cancer evolution under various selective pressures in addition to metastatic colonization, HV subpopulations were assessed for survival in the presence of various chemotherapeutics commonly used to treat breast cancer. HV subpopulations derived from both parental populations exhibited increased survival in the presence of doxorubicin, paclitaxel, cyclophosphamide and 5-fluorouracil (Fig. 3i,j). The ability of HV subpopulations to generate phenotypic diversity, metastasize more efficiently, and survive under mechanistically diverse chemotherapeutic agents is consistent with a positive role for phenotypic diversification in cancer progression.

### Nuclear size variation is associated with metastatic disease

**Figure 4:**
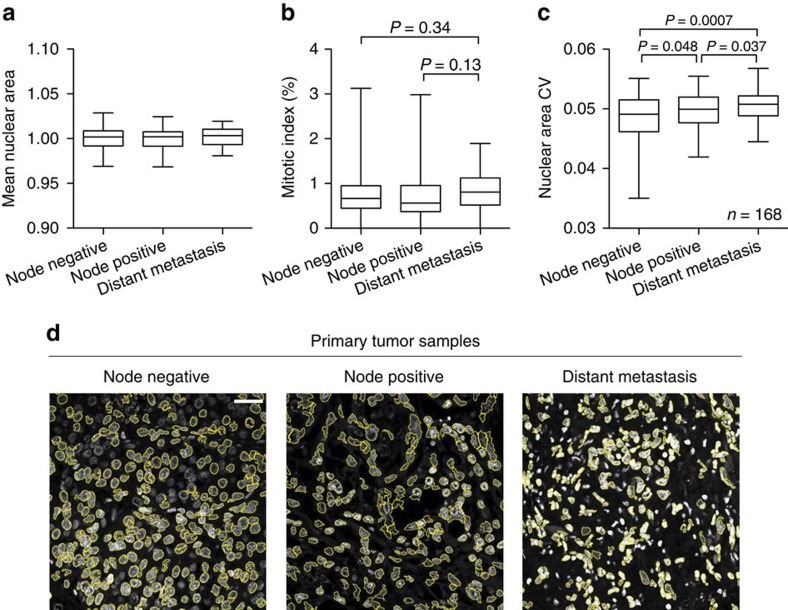
Nuclear area variability associates with breast cancer metastasis. (**a**–**c**) NCI CDP Breast Cancer Progression Tissue Microarray was stained with DAPI, imaged by confocal microscopy, and analysed for mean nuclear area (**a**), mitotic index (**b**) and nuclear area coefficient of variation (**c**) for each breast cancer core. Median, interquartile range, minimum, and maximum are depicted by box plots. *P*-values were derived using one-sided *t*-test. (**d**) Representative images with measured cancer cell nuclei (yellow boundary) are shown; scale bar, 50 um.

Given our observation of increased nuclear size variability in HV subpopulations (Supplementary Fig. 3a), we used this tractably quantifiable parameter as a readout of phenotypic variability in human invasive breast cancer tumour core biopsies. Mean nuclear size and mitotic index did not significantly correlate with disease stage (Fig. 4a,b). Consistent with our findings, nuclear area variability of cancer cells was significantly increased in tumours of patients with more advanced stage disease (Fig. 4c,d, Supplementary Fig. 3b). Primary tumours that progressed to lymph nodes displayed significantly higher variation in nuclear area than lymph node negative tumours. Furthermore, primary tumours that progressed to distant metastases exhibited significantly higher nuclear area variation than those tumours that did not metastasize. We also observed modestly higher, though not statistically significant, nuclear size variation in tumours with higher grade (Supplementary Fig. 3c), consistent with histopathologic observations^19^. These clinical correlations are consistent with our findings that cancer populations with greater diversification potential positively contribute to metastatic disease.

### Genomic and transcriptomic analysis of subpopulations

**Figure 5:**
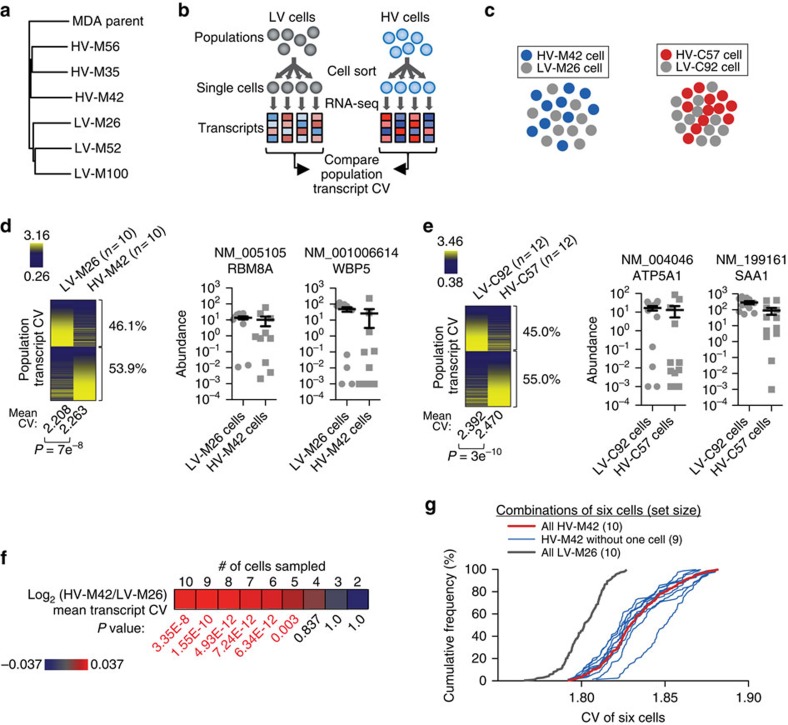
Highly variable populations exhibit increased cell-to-cell transcript expression variability. (**a**) Phylogenic tree based on genomic variant allele frequencies as measured by exome sequencing from cell populations. (**b**) Schematic for single cell sequencing and transcriptomic variability analysis. (**c**) Visualization of transcript expression profiles of 10 cells from selected MDA-derived subpopulations (left) and 12 cells from selected CN-derived subpopulations using t-distributed stochastic neighbor embedding (t-SNE). (**d**,**e**) Transcriptomic variability analysis was performed by calculating expression coefficient of variation for each transcript between cells from MDA-derived subpopulations (**d**; *n*=10 single cells per population) and from CN-derived subpopulations (**e**; *n*=12 single cells per population). Mean transcript coefficient of variation was calculated for all transcripts. *P* value was derived from two-sided paired *t*-test. Percentages of transcripts with higher CV in each population are shown. Representative highly variable transcripts in single cells are shown (right); *n*=10 single cells for MDA-derived populations and *n*=12 single cells for CN-derived populations. (**f**) Log-ratios of transcript CV mean were calculated by sampling MDA HV or LV subpopulations 10 times using different set sizes and calculating the mean transcript CV for HV and LV subpopulations. *P* values were derived using one-sided paired *t*-test. Highly significant *P*-values are in red. Scale bars are shown. All transcripts were used for analysis. (**g**) Cumulative frequency distribution is shown from sampling analysis. All possible combinations of six cells were sampled from the 10 MDA-derived HV cells with one specific cell removed (set size of 9) and distribution of mean CV is plotted (blue, 10 possible combinations to exclude each of the 10 cells). Distributions of mean CV from samplings of six cells from all HV cells (red, one possible combination to include all cells) and all LV cells (grey, one possible combination to include all cells) are shown. Error bars indicate s.e.m.

These clonal subpopulations of cells derived from isogenic backgrounds offer the potential for studies into the molecular basis of tumour heterogeneity. Given the contribution of genomic instability to tumour heterogeneity^20^,^21^, HV subpopulations were assessed for mutational burden. We quantified single-nucleotide variant and insertion–deletion mutation frequencies from population exome sequencing to reconstruct phylogenic relationships^1^. HV subpopulations appeared to diverge from a common HV ancestor and to exhibit genetic similarity to the parental population (Fig. 5a, Supplementary Fig. 4a,b), indicating that substantial genetic mutational changes did not accumulate as they clonally expanded. Furthermore, HV subpopulations did not significantly differ in the number of population-specific nucleic acid variants relative to LV subpopulations or the parental population (Supplementary Fig. 4c). Analysis of DNA content revealed no increase in mean DNA content or DNA content variability in HV subpopulations, suggesting against aneuploidy as the source of cell-to-cell variability (Supplementary Fig. 4d,e). While it is difficult to exclude the contribution of genetic alterations to HV subpopulation variability, the comparable genetic phenotypes and relative genetic stability in the isogenic HV subpopulations suggests that enhanced genetic diversification may not be the primary source of the observed phenotypic diversity.

### HV subpopulations display transcriptomic variability

To identify additional molecular mechanisms that could contribute to phenotypic diversity, single-cell RNA-sequencing from HV and LV subpopulations was performed to assess gene expression heterogeneity between cells (Fig. 5b). Assessment of single-cell RNA-sequencing fidelity demonstrated appropriate spike-in expression and no difference in spike-in variability between wells (Supplementary Fig. 5a,b). Total transcript abundance per cell showed no significant differences between HV and LV subpopulations (Supplementary Fig. 5c). Moreover, global mean transcript abundance was also not significantly different between HV and LV subpopulations (*P*=0.91 for HV-C57 versus LV-C92 and *P*=0.62 for HV-M42 versus LV-M26 by two-sided paired *t*-tests). Interestingly, linear and non-linear clustering^22^ of single-cell transcript expression profiles could not classify HV or LV subpopulations (Fig. 5c, Supplementary Fig. 5d), suggesting that averaged over-expression or repression of a gene or gene set may not be the primary cause of phenotypic variation recurrently observed in HV subpopulations^23^. However, global cell-to-cell transcript expression variability, as assessed by quantification of 8,218 transcripts from single MDA HV cells, was significantly elevated relative to expression variability between single LV cells (53.9% transcripts with higher CV in HV-M42; *P*=7e^−8^ by two-sided paired *t*-test; Fig. 5d). The same was observed for the HV-C57 subpopulation (5,826 transcripts; 55.0% transcripts with higher CV in HV-C57; *P*=3e^−10^ by two-sided paired *t*-test; Fig. 5e). Importantly, while an increased CV could occur by a decrease in mean expression, transcript variability observed in HV cells did not appear to be a byproduct of lowly expressed transcripts (Supplementary Fig. 5e,f).

To assess the robustness of this molecular phenotype, we determined whether sampling parameters of the single-cell sequencing experiments affected the observations. While more abundant transcripts are known to be detected with higher accuracy, elevated transcriptomic variability was observed regardless of transcript abundance (Supplementary Fig. 6a). To determine whether sequencing from limited number of cells affected the outcome, random sampling of single cells from subpopulations was performed to determine whether the enhanced transcriptomic variability could be detected with fewer cells. Indeed, transcriptomic variability was observed when setting the analysis to as few as five cells per population (Fig. 5f, Supplementary Fig. 6b). In addition, to determine whether a unique cell was responsible for the molecular variability, sampling was performed to assess population transcriptomic variability following removal of each single HV cell sequenced. Transcriptomic variability was consistently detected regardless of which HV cell was excluded from the analysis (Fig. 5g, Supplementary Fig. 6c), indicative of robust and indiscriminate population-level variability. While the increased transcriptomic variability appears modest, this effect is consistently observed under different analysis parameters and in two subpopulations derived from independent breast cancer populations. Taken together, these findings indicate that phenotypically diverse metastatic cancer subpopulations maintain enhanced intra-clonal transcriptomic variability generation capacity.

### Transcriptomic variability is transmitted to proteins

**Figure 6:**
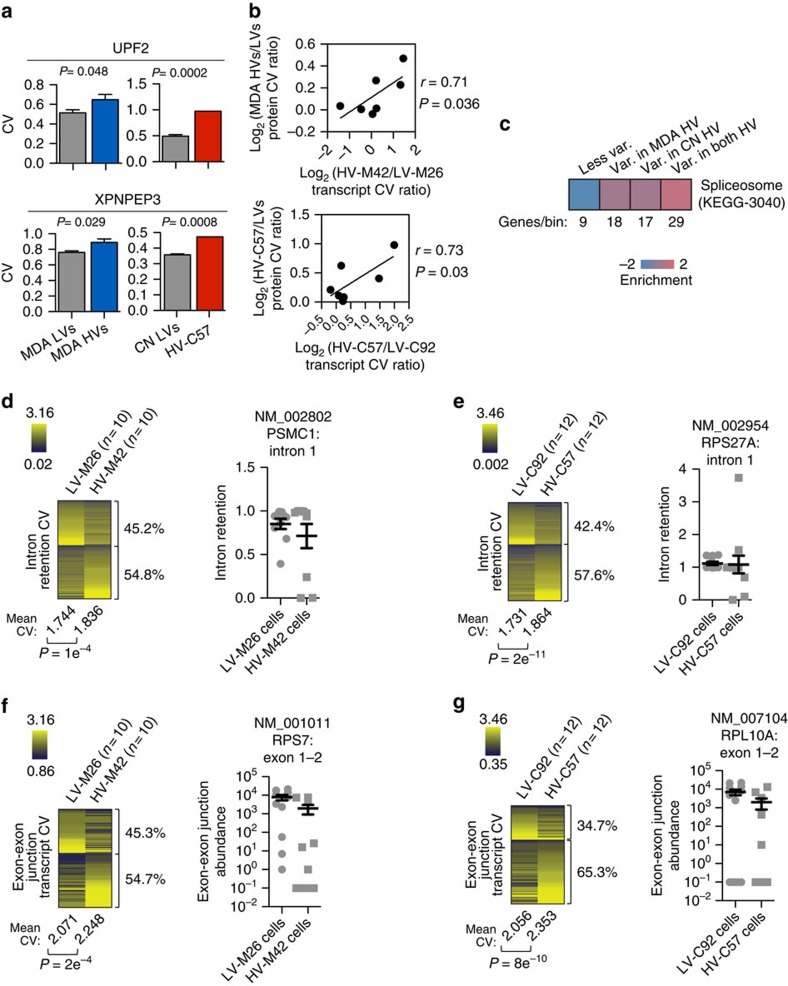
Cell-to-cell transcript expression variability is transmitted to the protein-level and is enriched for splicing factor components. (**a**) Flow cytometry was used to assess UPF2 (top) and XPNPEP3 (bottom) protein coefficient of variation in over 2.5 × 10^4^ cells. *P* values were derived using two-sample (MDA) or one-sample (CN) one-sided *t*-test. (**b**) Protein CV log-ratios of seven proteins as measured by flow cytometry from MDA-derived (top) and CN-derived (bottom) subpopulations was compared to transcript CV log-ratios measured by single-cell RNA-sequencing. *P* value was derived from testing Pearson's correlation coefficient with one-side. (**c**) Pathway analysis was used to identify gene sets that are enriched in transcripts with high expression variability in either or both HV subpopulations. Genes per bin and enrichment scale are shown. (**d**,**e**) Intron retention (IR) variability analysis was performed by calculating expression coefficient of variation for each IR measurement between cells from MDA-derived (**d**) and CN-derived (**e**) subpopulations. Mean IR coefficient of variation was calculated. *P*-value was derived from one-sided paired *t*-test. Percentages of retained introns with higher CV in each population are shown. Representative highly variable retained intron expression in single cells is shown (right); *n*=10 single cells for MDA-derived populations and *n*=12 single cells for CN-derived populations. (**f**,**g**) Exon–exon junction transcript variability analysis was performed by calculating expression coefficient of variation for each exon-exon junction transcript between cells from MDA-derived (**f**) and CN-derived (**g**) subpopulations. Mean exon-exon junction transcript coefficient of variation was calculated. *P* value was derived from one-sided paired *t*-test. Percentages of exon-exon junction transcripts with higher CV in each population are shown. Representative highly variable exon-exon junction transcript expression in single cells is shown (right); *n*=10 single cells for MDA-derived populations and *n*=12 single cells for CN-derived populations. Error bars indicate s.e.m.

To validate the relevance of transcriptomic variability as it pertains to biological function, we used flow cytometry to assess protein-level variation. *XPNPEP3* and *UPF2*, two genes that displayed high transcript-level variability in both HV subpopulations (Supplementary Table 1) and whose protein expression per cell could be readily quantified in a high-throughput manner by flow cytometry, demonstrated a consistent level of increased protein expression variability in HV subpopulations (Fig. 6a). We extended these findings to the following additional five proteins that could be readily quantified by flow cytometry: ALDOA, PABPC1, HNRNPA1, CD110, and HNRNPA0. In all HV and LV subpopulations, we observed a significant correlation between transcript-level variation and protein-level variation for the seven genes tested (Fig. 6b) with no consistent correlation in mean protein abundance (Supplementary Fig. 7a). These findings reveal that transcript variation is transmitted to the protein level and highlight high-molecular variability as a principal feature of HV subpopulations.

### Spliceosomal gene transcripts are highly variable

We next sought to identify a potential mechanism that could contribute to transcriptomic variation in highly variable subpopulations. MDA-derived and CN-derived subpopulations displayed similarity in highly variable genes (Supplementary Table 1), many of which alone could contribute to global transcriptomic variability such as chromatin modifiers (SENP7, ARID1A), transcription factors (TCF7L2, SP3), and regulators of non-sense-mediated decay (UPF2). While individual genes most likely contribute to the effect seen in highly variable populations, we hypothesized that coordinated variation of a common subset of transcripts may reveal a major contribution to cell-to-cell variability. To identify regulatory networks that might be responsible for transcriptomic variation, we searched for functional gene sets that exhibited high-transcript expression variability. Transcripts were binned into four categories depending on whether relative transcript variability was increased in either or both MDA-derived and CN-derived HV subpopulations. Pathway discovery analysis (iPAGE^24^) revealed spliceosome machinery and myeloid cell differentiation gene transcripts as the only two gene sets to exhibit significantly higher variability in HV subpopulations derived from both patients' cancer populations (Fig. 6c, Supplementary Fig. 7b). This raised the possibility that cell-to-cell variation in the expression levels of splicing genes and the resultant mRNA processing activity may represent a conceivable mechanism through which population-level heterogeneity of mature transcripts may be achieved at a global scale^25^.

Improper splicing may lead to an abundance of unspliced pre-mRNAs, which are eventually degraded via non-sense-mediated decay^26^. Inefficient splicing could thus reduce the expression of a large number of mature transcripts. Given the expression variation of spliceosome machinery components, we reasoned that variation in constitutive spliceosome activity in HV cells may cause enhanced variation in global unspliced pre-mRNA levels. Variation in unspliced pre-mRNA levels could then contribute to transcript abundance variation. Indeed, HV subpopulations demonstrated higher cell-to-cell unspliced pre-mRNA variability, as determined by the analysis of 1,132 matched retained introns from MDA-derived cells (54.8% retained introns with higher CV in HV-M42, *P*=1e^−4^ by one-sided paired *t*-test; Fig. 6d) and 1,666 matched retained introns from CN-derived cells (57.6% retained introns with higher CV in HV-C57, *P*=2e^−11^ by one-sided paired *t*-test; Fig. 6e). In addition, if this unspliced pre-mRNA variability as measured by retained introns is propagated, it should be apparent in further processed forms of pre-mRNAs. Indeed, exon–exon junction expression variability measured from single-cell sequencing experiments was significantly increased in HV subpopulations from MDA-derived cells (54.7% exon–exon junctions with higher CV in HV-M42, *P*=2e^−4^ by one-sided paired *t*-test; Fig. 6f) and CN-derived cells (65.3% exon-exon junctions with higher CV in HV-C57, *P*=8e^−10^ by one-sided paired *t*-test; Fig. 6g). This molecular variability could be caused by variation in splicing as well as varied decay of improperly spliced transcripts. Consistent with our findings, intron retention has been previously observed to be the most significant splicing alteration in breast cancer patient samples^27^. These findings reveal enhanced splicing variability as one feature of highly variable subpopulations.

### Variability in SNRNP40 expression enhances metastasis

**Figure 7:**
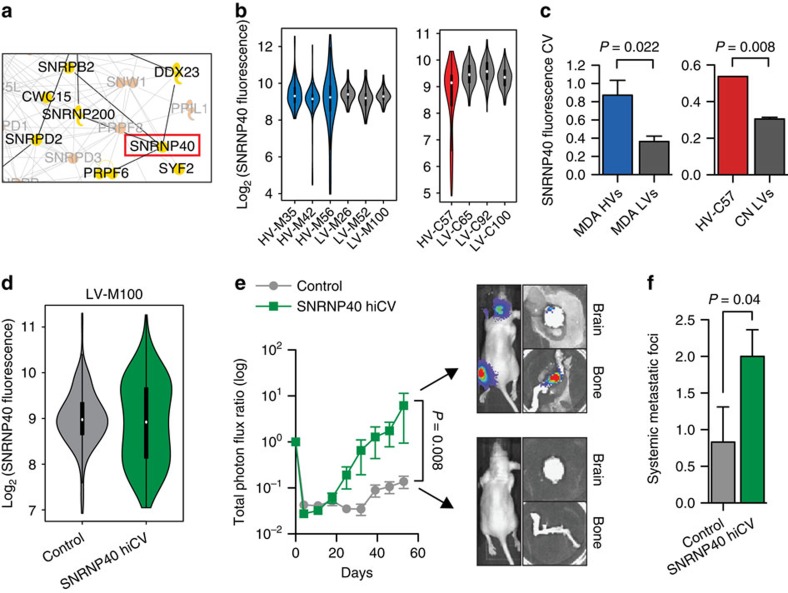
High expression variability of spliceosomal gene SNRNP40 leads to increased metastatic capacity in low variability population. (**a**) Ingenuity Pathway Analysis was used to identify spliceosome gene interactions. Genes in yellow displayed increased variability in both HV subpopulations, while genes in orange displayed increased variability in only one HV subpopulation. Black lines indicate annotated direct interactions of genes with increased variability in both HV subpopulations. Grey lines indicate interactions that are indirect or do not involve a gene with increased variability in both HV subpopulations. (**b**,**c**) Subpopulations were quantified for SNRNP40 protein levels by fluorescence confocal microscopy (**b**) and analysed for protein expression coefficient of variation (**c**). *P*-values were derived from two-sample (MDA) or one-sample (CN) one-sided *t*-test. (**d**) SNRNP40 protein expression of control and high SNRNP40 expression variability pooled LV-M100 populations as measured by fluorescence microscopy. (**e**) 10^5^ cells from pooled LV-M100 populations were inoculated via intracardiac injection and monitored by bioluminescence flux. Representative mice are shown. *P* value was derived from one-sided *t*-test; *n*=6. (**f**) Systemic metastatic foci per mice were counted as distinct, minimal bioluminescence signals. *P*-value was derived using one-sided *t*-test. Error bars indicate s.e.m.

Finally, we sought to determine whether enforced modulation of a highly variable spliceosomal gene could recapitulate the metastatic capacity seen with HV subpopulations. While highly variable subpopulations displayed variability in many spliceosomal genes, focused study on a single spliceosomal gene could serve as a model by which variation of other spliceosomal genes may contribute to expression variation. However, focus on a single gene would be expected to recapitulate only a fraction of the effects seen in highly variable subpopulations. We focused on SNRNP40, a component of the U5 small nuclear RNP complex, because this gene exhibited high transcript variability in HV subpopulations (Supplementary Fig. 8a), was among the top three most variable spliceosomal gene transcripts (Supplementary Table 2), has been described to directly interact with numerous highly variable spliceosomal genes (Fig. 7a, Supplementary Table 2), and its protein expression could be readily quantified by immunofluorescence-based imaging (Supplementary Fig. 8b). Indeed, HV subpopulations displayed increased SNRNP40 protein-level variability (Fig. 7b,c). To determine whether population-level variation in SNRNP40 observed with HV cells was sufficient to enforce metastatic fitness, LV subpopulations were generated to express varying levels of SNRNP40 (Supplementary Fig. 9a). These populations were pooled to generate a mixed population with increased cell-to-cell SNRNP40 expression variation without significantly altering mean SNRNP40 expression (Fig. 7d; mean: Control=9.0, hiCV=8.9, *P*=0.19 by two-sided *t*-test; CV: Control=0.068, hiCV=0.104, *P*=7e-14 by Levene's test). Functional testing of this engineered population revealed that increased cell-to-cell variability in SNRNP40 expression enhanced the ability of these cells to metastasize more efficiently and to colonize more sites systemically (Fig. 7e,f), suggesting that variation in SNRNP40 between cells could, in part, contribute to enhanced metastatic capacity observed in HV subpopulations.

**Figure 8:**
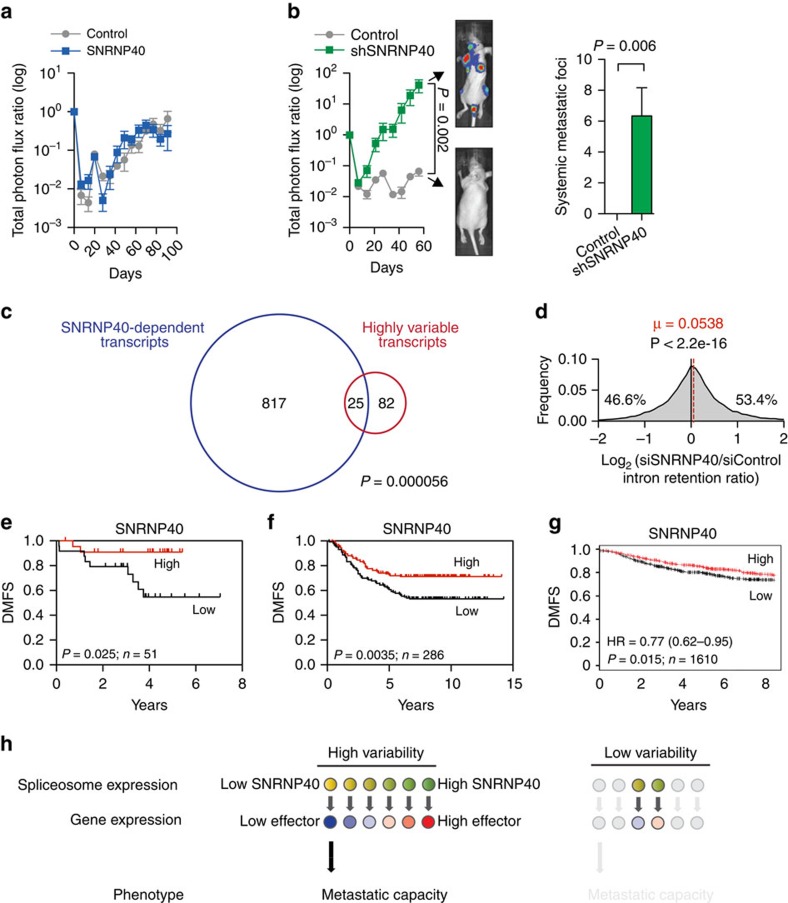
Low SNRNP40 expression increases metastatic colonization capacity and is associated with metastatic outcomes in patients. (**a**) 10^5^ LV-M100 cells with SNRNP40 over-expression were inoculated by intracardiac injection and monitored by bioluminescence; *n*=5. Error bars indicate s.e.m. (**b**) 10^5^ LV-M100 cells with SNRNP40 knockdown were inoculated by intracardiac injection and monitored by bioluminescence. Representative mice are shown; *n*=5. Systemic metastatic foci per mice were counted (right). *P*-values were derived from one-sided *t*-test. Error bars indicate s.e.m. (**c**,**d**) LV-M100 cells were transfected with two siRNAs targeting SNRNP40 and two control siRNAs and processed for gene expression analysis. (**c**) Venn diagram showing the overlap between SNRNP40-dependent transcripts (absolute log-foldchange >1 on siRNA treatment) and highly variable transcripts from the MDA single cell experiment (transcript CV ratio >1.5). *P*-value is derived from hypergeometric distribution. (**d**) Log-foldretained intron ratio was calculated for each transcript and plotted as a histogram. Dashed red line indicates mean. Percentages of retained intron ratios above and below zero are shown. *P*-value was derived from two-sided *t*-test of log-ratio as compared with zero. (**e**–**g**) Kaplan–Meier curves of distant metastasis-free survival (DMFS) from GSE33926 (ref. 43) (**e**), GSE2034 (ref. 42) (**f**) and kmplotter meta-analysis^44^ (**g**) as a function of primary tumour SNRNP40 expression in breast cancer. *P*-values were derived from log-rank test. (**h**) Model of spliceosome expression variability mediating gene expression changes and metastatic capacity heterogeneity.

We sought to determine whether within this cell population engineered to express higher SNRNP40 variation, the cells with high SNRNP40 or those with low SNRNP40 levels imparted the enhanced fitness to the pooled population. To do this, we generated cells that represented the tails of the variable population—those that expressed high SNRNP40 through over-expression, or those that expressed low SNRNP40 through knockdown. While increased SNRNP40 expression did not affect metastatic capacity (Fig. 8a), SNRNP40 depletion significantly promoted systemic metastasis (Fig. 8b, Supplementary Fig. 9b–e). Next, we sought to determine whether variation in SNRNP40 expression contributes to the gene expression variability seen in highly variable subpopulations. Our model is that variable levels of SNRNP40 allows for the transmission of variability to many transcripts dependent on SNRNP40 expression. Thus, we used small interfering RNA (siRNA)-mediated depletion of SNRNP40 to identify these dependent transcripts and inferred a high magnitude of expression change to be indicative of strong transmission. If variable expression of SNRNP40 contributes to gene expression variability seen in HV subpopulations, SNRNP40-dependent transcripts would be enriched among variable transcripts in HV subpopulations. To test this hypothesis, the degree of overlap between SNRNP40-dependent transcripts (absolute log foldchange >1 on siRNA treatment) and highly variable transcripts (transcript CV ratio from MDA single-cell sequencing >1.5) was assessed. Indeed, transcripts with high magnitude expression change in SNRNP40 knockdown cells were significantly enriched among variable transcripts in HV single cells (Fig. 8c). This enrichment can also be demonstrated as a heat map (Supplementary Fig. 10a) and as a binned dot plot, which shows that the degree of gene expression deregulation on SNRNP40 depletion significantly correlated with transcript variability in the HV population (Supplementary Fig. 10b). These findings suggest that SNRNP40 contributes in part to the expression of a set of variable transcripts in breast cancer cells. In addition, SNRNP40 depletion significantly increased the fraction of unspliced pre-mRNAs (Fig. 8d), consistent with pre-mRNA variability observed in HV subpopulations. The expected modest effect is likely attributable to perturbation of only a single spliceosomal gene. Consistent with these functional findings, decreased SNRNP40 transcript-level expression in bulk primary breast cancer samples was associated with increased metastatic relapse outcomes in multiple independent data sets (Fig. 8e–g). These findings provide clinical association support for SNRNP40 expression in human breast cancer progression. While tumours can achieve reduced SNRNP40 expression through mean expression alteration, we show in our experimental model that cancer subpopulations may achieve SNRNP40 silencing through deregulation in a subset of cells that may not be apparent from averaged measurements derived from bulk tumours.

## Discussion

Previous studies have revealed the benefit of non-genetic cell-to-cell variability to fitness of cells under cytotoxic conditions^4^,^8^,^28^,^29^. We show that enhanced variation at the transcriptomic level is generated and maintained within rare clonal cancer subpopulations, leading to phenotypic diversity and enhanced metastatic capacity. These HV cells were isolated from parental populations at a low frequency of 1–3%, which could be attributed to a number of factors. First, the increased variability of HV cells that leads to increased fitness *in vivo* may not be beneficial *in vitro* where the cell culture selective pressures may be more suitable for LV cells. Similarly, phenotypic trade-offs at the cellular level might contribute to the low frequency of HV cells^17^; for example, if beneficial diversity were hypothetically generated through an energetically demanding and slower cell division process, proliferation rate would be sacrificed as a result. Aside from potential cell-intrinsic mechanisms, clonal interactions within the parental population are likely involved in maintaining the presence of HV cells at a low frequency. Subclonal heterogeneity can be maintained through subclonal cooperation from both minor subpopulations that enhance proliferation of neighbouring cells as well as other populations that contribute to tumour growth^30^. HV cells might similarly utilize non-cell-autonomous mechanisms to maintain variation under *in vitro* conditions with other cells in the population more suited for growth in culture.

We consistently observe molecular variability in HV subpopulations by different experimental assays and at both the molecular and phenotypic levels. Molecular and phenotypic variation within cancer populations increases the likelihood that an individual cell is able to survive and repopulate a tumour under a given selection pressure, such as metastatic colonization and chemotherapy. We propose spliceosome-associated gene expression variability as one mechanism by which clonal cancer populations could increase mature transcript expression variability of target genes. We show one example of spliceosome gene expression variability where variable cell-to-cell expression of SNRNP40 enables enhanced metastatic fitness through generation of a cell subpopulation with reduced SNRNP40 expression (Fig. 8h). This mechanism is supported by the requirement of key splicing factors for maintenance of robust transcriptomes^31^. Minimal deregulation of multiple splicing factors has the potential for amplified alterations of gene regulatory networks and gene expression states. In addition, intron retention has been observed to regulate expression of genes involved in nuclear shape as well as splicing factor genes^26^. The consistent molecular variability observed in HV subpopulations derived from independent human cancer cell populations suggests that deregulation of specific sets of downstream targets is molecularly conserved. These experimental observations can be tested in clinical correlates to characterize non-genetic contributions to tumour evolution in patients. While the upstream cause of the transcript-level variability remains to be determined, we propose deregulated population transcriptomic variability to represent one mechanism by which molecular diversity can be achieved in cancer and reveal enhanced phenotypic diversification capacity to associate with metastatic progression.

## Methods

### Cell culture

MDA-MB-231 cells and their derivatives were maintained in DMEM supplemented with 10% FBS, glutamine, pyruvate, penicillin, streptomycin and fungizone. CN34 cells and their derivatives were maintained in M199 supplemented with 2.5% FBS, 10 μg ml^−1^ insulin, 0.5 μg ml^−1^ hydrocortisone, 20 ng ml^−1^ EGF, 100 ng ml^−1^ cholera toxin, glutamine, pyruvate, penicillin, streptomycin and fungizone. MDA-MB-231 and CN34, cell lines were originally obtained from ATCC, while CN34 cell lines were generated from pleural fluid of a breast cancer patient as previously described^32^. Cells in culture were routinely tested for mycoplasma contamination. Clonal populations were generated from parental populations by seeding cells sparsely, picking individual colonies, and expanding to ∼10^5^ cells when cells were imaged for size measurements.

### Cell size measurements and coefficient of variation analysis

3 × 10^4^ cells were seeded on coverslips and stained with HCS CellMask Red (Invitrogen) and DAPI. Numerous fields were imaged on DeltaVision Image Restoration Microscope to capture at least 100 cells per population. Cell size parameters were measured using Cell Profiler 2.0. Debris was filtered out by generating a histogram of (cytoplasmic area—nuclear area) and applying a minimum threshold in R. To calculate coefficient of variation (s.d./mean) for each clonal population, a sampling size was determined from the clonal population with the fewest cells analysed (96 cells in the MDA-MB-231 population and 206 cells in the CN34 population). Subpopulations with more cells imaged than the sampling size were sampled 100 times, with the average CV used in the final analysis. For each subpopulation, coefficient of variation was calculated for each size parameter, which includes cell area, cytoplasmic area, nucleus, perimeter, major axis length, and minor axis length. Principal component analysis was performed using all subpopulations on the coefficient of variations for all morphological parameters. The first principal component accounted for 93 and 91% of the variance in MDA and CN34 cell lines, respectively, and is used for all size variation analyses.

### Three-dimensional size coefficient of variation analysis

Cells were fixed in 8% paraformaldehyde, permeabilized with 0.1% Triton-X, and stained with CellMask Red. Cells were analysed by ImageStream-X (Amnis) to measure cell size, nuclear size, perimeter, major axis and minor axis. Principal component analysis was performed, and the first principal component was used for analyses.

### Proliferation & colony formation assays

For proliferation assays, 5 × 10^3^ cells were seeded in triplicate and assayed WST-1 reagent (Roche) 72 h after seeding. For colony formation assays, 200 cells were seeded in 10 cm plates in triplicate and were allowed to grow for 10 days (MDA-derived cells) or 20 days (CN-derived cells). Plates were fixed in 6% glutaraldehyde with 0.5% crystal violet and scanned. Colony areas were measured using ImageJ. R was used to remove debris, equalize colony numbers with samplings as above, and calculate coefficient of variation.

### Animal studies

Animal experiments were conducted in accordance with protocols approved by the Institutional Animal Care and Use Committee at The Rockefeller University. For *in vivo* experiments with MDA-derived cell lines, populations were transduced with a retroviral construct expressing a luciferase reporter^33^. CN34 parental cell line was previously labelled with luciferase reporter. Athymic female mice aged 6 weeks (Jackson labs) were used for intracardiac injection. NOD-SCID female mice aged 6 weeks (Jackson labs) were used for tail vein injections with MDA-derived cells, while NOD-SCID gamma female mice aged 6 weeks (Jackson labs) were used for tail vein injections with CN-derived cells. Sample size in mice experiments was chosen based on biological variability observed with a given genotype. Animals were excluded from studies if inoculated cells did not arrive in the intended site. For portal circulation injections, cells were injected into the spleen of NOD-SCID gamma female mice followed by removal of the spleen. For portal circulation injection of mixed population, LV-M100 cells were transduced with pLKO.1 puro (Addgene plasmid #8453), and HV-M42 cells were transduced with pLKO.1 blast (Addgene plasmid #26655). Cells were mixed at a 1:1 ratio immediately before injections. *In vivo* bioluminescence was monitored weekly by retro-orbital injection of luciferin (Perkin Elmer) and normalized to bioluminescence signal immediately following cell injection.

### Tissue microarray analysis

NCI CDP Breast Cancer Progression Tissue Microarray slides were obtained from the Cancer Diagnosis Program at the National Cancer Institute, US National Institutes of Health. Tumour cores were obtained from The Cooperative Breast Cancer Tissue Resource with informed consent and ethical approval as indicated^34^. TMA slides were deparaffinized, rehydrated, and exposed to Heat Induced Epitope Retrieval at pH 6. Slides were stained with DAPI, and tumour cores were imaged on Leica TCS SP5 system at × 40. Images were analysed using CellProlifer 2.0 to identify and measure cancer cell nuclei by Otsu Adaptive thresholding. Image acquisition and analysis were blinded until measurements were completed for all tumour cores. Mitotic index was calculated as follows: number of mitotic cells/total cells for each tumour core.

### Exome sequencing and analysis

gDNA was extracted using DNEasy kit (Qiagen). Libraries were prepared using Nextera Extended Exome sequencing kit, as per manufacturer's instructions (Illumina), and paired-end sequenced on HiSeq 2500 (Illumina). The analysis pipeline for the exome-seq data was based on the GATK best practices. Reads were aligned to the human genome (build hg19). The paired-end reads were then fixed and filtered using Picard (v. 1.107; http://picard.sourceforge.net/). The duplicates were also removed in the same step. Using GATK (v. 2.5)^35^, the reads were realigned and recalibrated. mpileup (samtools^36^) was used to create an input for VarScan (v2.3.6)^37^. In VarScan, mpileup2snp and mpileup2indel commands were used to identify variants across all exome-seq samples. To study population genetic divergence, the frequencies of all variants identified (148,234) were used to generate phylogenic tree by Nei's genetic distance using neighbor-joining method in the PHYLIP package.

### Single cell RNA-sequencing

Single cell isolation, cDNA synthesis, amplification, and processing for Illumina sequencing were performed as described^38^. Reads were distributed into separate samples based on their barcodes. Cells were excluded if wells were empty or generated library was low quality as assessed by the number of total reads, number of mapped reads, a high percentage of unmapped reads and a high percentage of spike-in reads^39^. To equalize the number of cells analysed from each population, cells were randomly selected and removed from analysis. The RNA-seq pipeline described above was then used to measure gene expression across RNA-seq data from each cell. In parallel, TopHat results were parsed to count the presence of every exon-exon junction across all the samples. Transcripts present in both cell populations, present in more than 25% of all cells and expressed above threshold mean of all cells based on a Gaussian distribution were included for analysis. Non-linear cell clustering by t-Distributed Stochastic Neighbor Embedding was performed using ‘tsne' package in R. To measure retained introns, the number of reads mapping to each exon or intron were counted across the transcriptome for all samples. As a measure of intron retention (IR), for each intron, we then calculated the number of reads mapping to each intron relative to the two spanning exons (r_int_ex). For CV measurements, retained introns and exon-exon junctions were included in analysis if present in more than 25% of all cells.

### Pathway analysis

iPAGE^24^ pathway analysis (http://iget.c2b2.columbia.edu) was used to identify gene sets with higher transcript variability in both HV subpopulations. Transcript coefficient of variation log-ratios (HV/LV) were used to categorize transcripts into four bins: bin 1) negative log-ratio in both MDA-derived and CN-derived comparisons, bins 2 and 3) positive log-ratio in either MDA-derived or CN-derived comparison, and bin 4) positive log-ratio in both MDA-derived and CN-derived comparisons. GO and KEGG annotations were analysed using a maximum *P*-value of 0.05 and maximum genes per category of 200. Gene sets were only considered relevant if enrichment was highest in bin 4 and lowest in bin 1. Ingenuity Pathway Analysis (Qiagen) was used to identify spliceosomal gene interactions based on published literature.

### Flow cytometry

For antibody staining, cells were prepared using Cytofix/Cytoperm (BD). Dead cells were excluded using Live/DEAD Aqua (Invitrogen). Primary antibodies used were anti-XPNPEP3 (Abcam 25D, 1:200), anti-UPF2 (LSBio LS-C160443, 1:4) anti-ALDOA (Abcam ab54770, 1:8), anti-PABP (Abcam 10E10; 1:200), anti-HNRNPA1 (Cell Signalling D21H11, 1:30), anti-CD110 (BD clone 1.6.1, 1:50), anti-HNRNPA0 (Cell Signalling D8A3, 1:50), anti-ESR1 (Thermo MA1310, 1:20), anti-MCF2 (LSBio LS-C164083, 1:10), and anti-CSF2RA (eBiosciences 4H1, 1:30) conjugated to Alexa555 or Alexa647 Zenon secondary antibodies (Invitrogen). FACS was performed on LSRII (BD). Analysis was performed on FloJo. CV calculations were performed using greater than 25 × 10^3^ cells, an equal number of cells per sample within each experiment. Ratios were calculated using the average value of all HV populations (MDA: HV-M35, HV-M42, HV-M56; CN: HV-C57) and LV populations (MDA: LV-M26, LV-M52, LV-M100; CN: LV-C65, LV-C92, LV-C100). Cell cycle analysis was performed by employing flow cytometry on fixed cells stained with DAPI and determining cell cycle phases in FloJo.

### SNRNP40 protein quantitation

Cells were fixed, permeabilized and stained with DAPI and HPA026527 (1:100, Sigma) followed by fluorescent-conjugated secondary antibodies. Imaging was performed on Leica TCS SP5 system. SNRNP40 relative protein level was determined using Cell Profiler 2.0 by measuring total nuclear SNRNP40 fluorescence intensity as demarcated by DAPI signal.

### SNRNP40 cell line generation

To generate high CV SNRNP40 population, cell populations were transduced individually at various titres with either virus for short hairpin RNA (shRNA) expression (shRNA#1 TRCN0000074608 and shRNA#2 TRCN0000074610, Sigma) or virus for stable ORF expression (pBabe vector). Expression in individual populations was confirmed by quantitative reverse transcriptase–PCR (RT–PCR). Populations were then pooled at equal ratios to generate mixed populations. Control population was generated similarly using a non-targeting shRNA (SHC016, Sigma) and an empty expression vector.

### Quantitative RT–PCR

RNA was extracted using total RNA isolation kit (Norgen Biotek). cDNA was generated using Superscript III (Invitrogen). Fast SYBR Green Master Mix (Life Technologies) was used to analyse samples on Applied Biosystems 7900HT. Expression was normalized to GAPDH. Primers sequences are as follows: SNRNP40 Forward: 5′-CAGTGGAGCAGTGATGGAAT-3′; SNRNP40 Reverse: CCCTCTCACCTGTTTCACTATC-3′; GAPDH Forward: 5′-AGCCACATCGCTCAGACAC-3′; GAPDH Reverse: 5′-GCCCAATACGACCAAATCC-3′; blasticidin Forward: 5′-CCTGGGATCAAAGCCATAGT-3′; blasticidin Reverse: 5′-TTAGCCCTCCCACACATAAC-3′; puromycin Forward: 5′-GTCACCGAGCTGCAAGAA-3′; puromycin Reverse: 5′-CCGATCTCGGCGAACAC-3′.

### siRNA transfection

The following siRNAs were used (IDT): siSNRNP40 #1: 5′-GGAAUAGACAAUGAUAUC-3′; siSNRNP40 #2: 5′-GGAUUUGACCGACUGAUA-3′. BLOCK-iT Fluorescent Oligo (Life Technologies) and NC1 (IDT) were used as controls.

10^5^ cells were seeded and were transfected the next day with siRNAs via Lipofectaime 2000 (Invitrogen). Cells were extracted 48 h later for validation of knockdown by quantitative RT–PCR, RNA-sequencing or flow cytometry. For RNA-sequencing, two siRNAs were used for SNRNP40 and control, and independent siRNA replicates were averaged.

### RNA-sequencing

RNA was extracted using total RNA isolation kit (Norgen Biotek) with DNAse I treatment followed by Ribo-Zero Gold rRNA removal (Epicentre). Libraries were generated using ScriptSeq v2 RNA-seq Library Preparation Kit (Epicentre) and run on HiSeq 2500. For RNA-seq data analysis, the reads were trimmed to remove matches to linkers and low-quality bases (cutadapt v1.2.1). Tophat (v. 2.0.8)^40^ was then used to map the reads to the human transcriptome (build hg19). Cufflinks and cuffmerge (v.2.0.2) were then used to calculate reads per kilo base per million and consolidated results across the samples. Finally, cuffdiff (v.2.0.2) was used to calculate log-foldchanges and the associated statistics. Enrichment analysis was performed as previously described^41^. Statistical test for Venn diagram overlap was performed using phyper() in R.

### Clinical association analyses

GSE2034 (ref. 42) and GSE33926 (ref. 43) were used to generate Kaplan–Meier curves. Patients were stratified by SNRNP40 expression relative to median. KMplotter was used to assess distant metastasis-free survival with follow up threshold of 8 years^44^.

## Additional information

**Accession codes:** Exome sequencing data has been deposited in the European Nucleotide Archive (ENA) under the accession code PRJEB12872. RNA-sequencing data have been deposited in the GSE under the accession code GSE78527.

**How to cite this article:** Nguyen, A. *et al*. Highly variable cancer subpopulations that exhibit enhanced transcriptome variability and metastatic fitness. *Nat. Commun.* 7:11246 doi: 10.1038/ncomms11246 (2016).

## Supplementary Material

Supplementary Information Supplementary Figures 1-10 and Supplementary Tables 1-2
