# Supplementary material for: Highly variable cancer subpopulations that exhibit enhanced transcriptome variability and metastatic fitness
Source: Nat Commun. 2016 May 3;7:11246. doi: 10.1038/ncomms11246 (PMC4857405; doi:10.1038/ncomms11246)
Supplement: Supplementary Information — Supplementary Figures 1-10 and Supplementary Tables 1-2 [file ncomms11246-s1.pdf]

Supplementary Figure 1

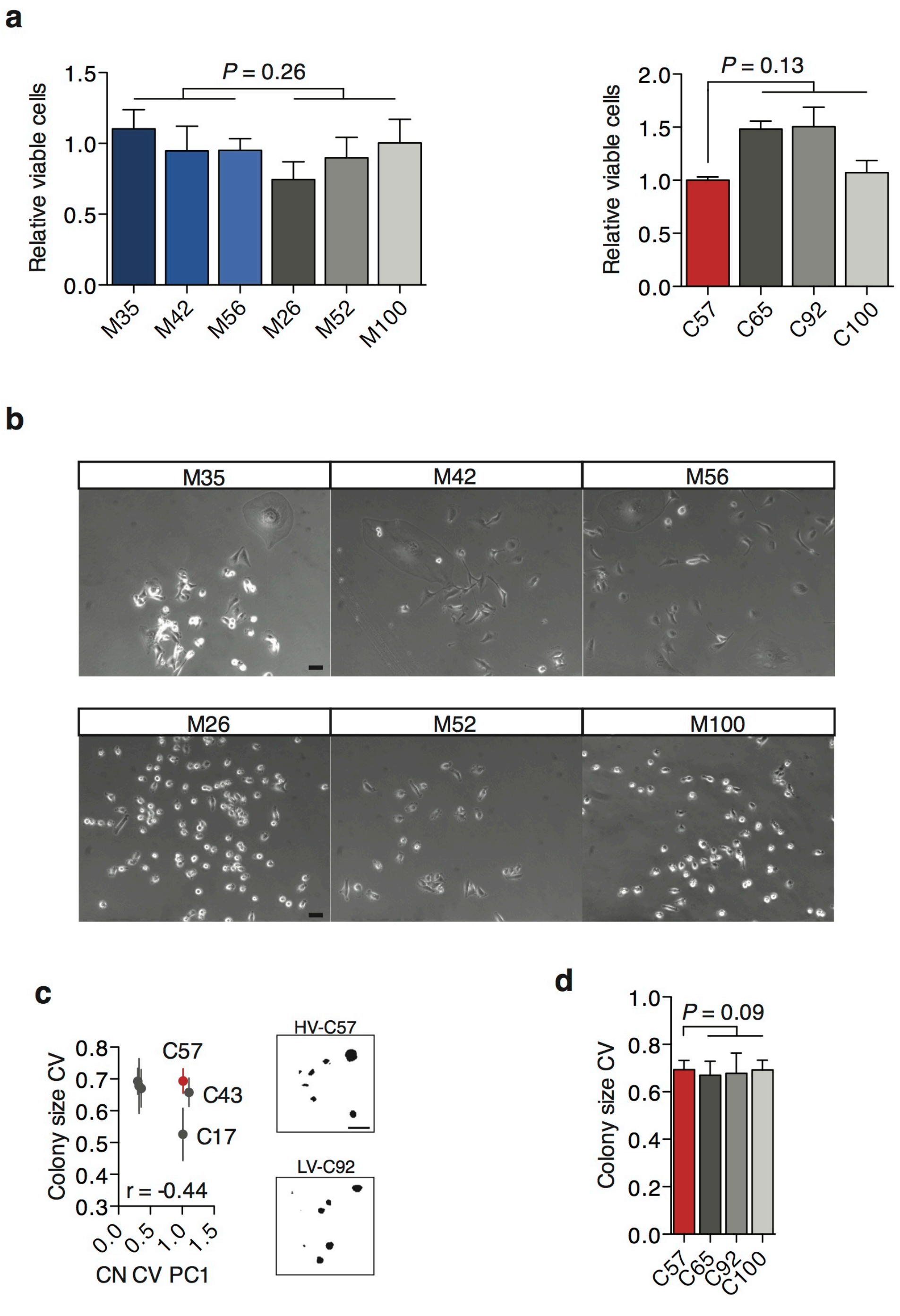

Supplementary Figure 1. Proliferation and colony size variation of clonal subpopulations

a, $5x10^3$ cells were seeded and assessed 72 hours later for viable cells using WST-1 reagent. P-values were derived from two-sided student's *t*-test (two-sample for MDA, one-sample for CN). Error bars indicate s.d. **b**, Representative images of cells in a colony from colony formation assay are shown; scale bar is 100um. **c**, CN-derived colonies were grown in triplicate plates and measured for variation in colony area. Error bars indicate s.e.m. of three independent experiments. Pearson's correlation coefficient with one-side is shown. HV subpopulation C57 is in red. Representative images of colonies are shown; scale bar is 5mm. **d**, Colony size coefficient of variation for selected subpopulations is shown. P-value was derived from one-sided one-sample student's *t*-test.

Supplementary Figure 2

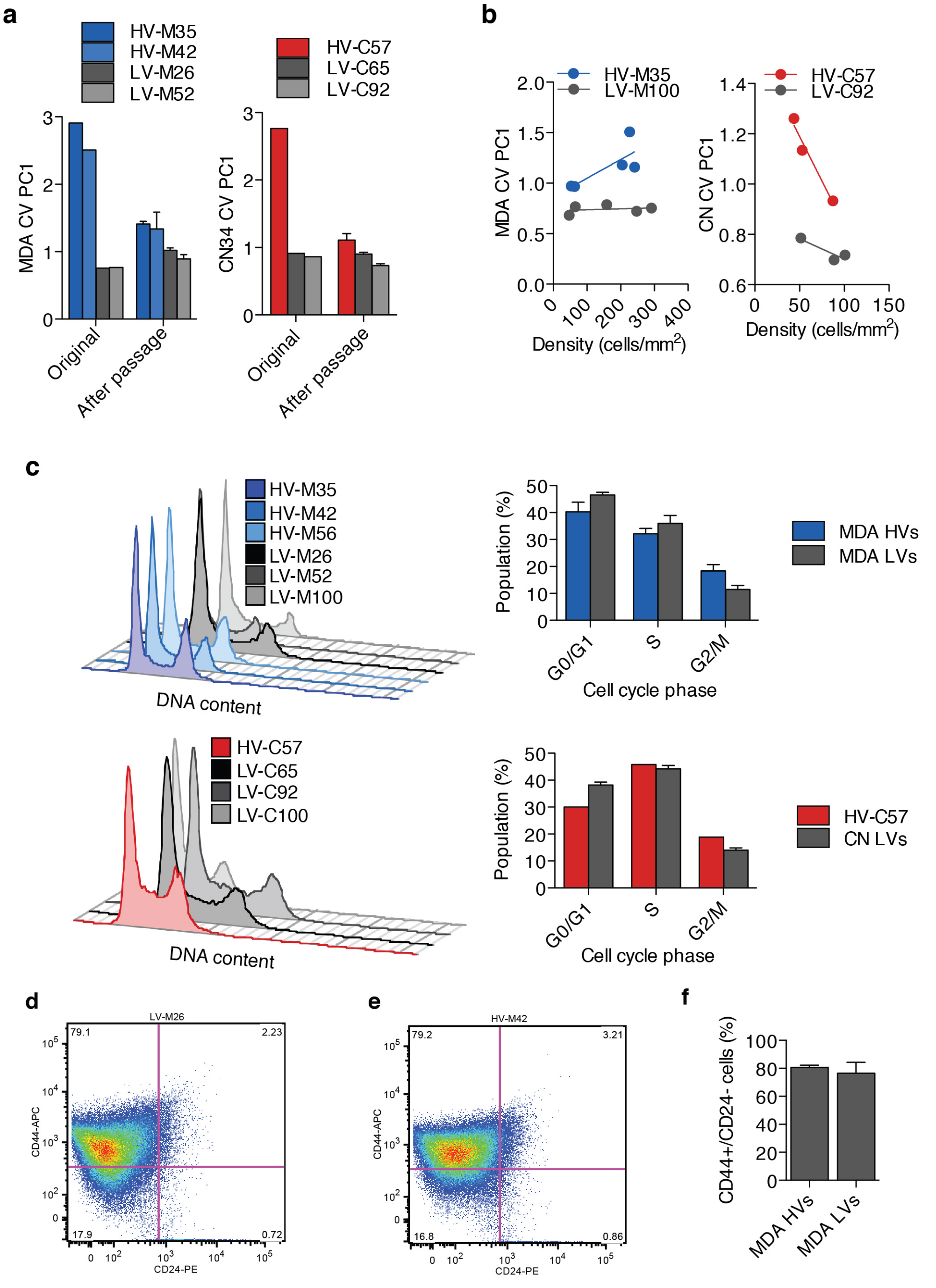

Supplementary Figure 2. Phenotypic characterization of highly variable populations

a, Populations were passaged five times in culture under standard cell culture conditions, then imaged and analyzed for cell size coefficient of variation. Error bars indicate s.d. **b**, Populations were seeded at various densities, then imaged and analyzed for cell size coefficient of variation. **c**, Fixed cells were stained with DAPI and analyzed by flow cytometry to measure DNA content. Histograms of each population are shown (left). Watson pragmatic cell cycle analysis was performed in FlowJo to assess cell cycle phasing (right). **d**, **e,** LV-M26 (**d**) and HV-M42 (**e**) subpopulations were assessed for expression of cell surface markers CD24 and CD44. **f**, Proportion of CD44+/CD24- cells in MDA HV subpopulations (n=3) and MDA LV subpopulations (n=3) was assessed. Error bars indicate s.e.m. unless otherwise indicated.

Supplementary Figure 3

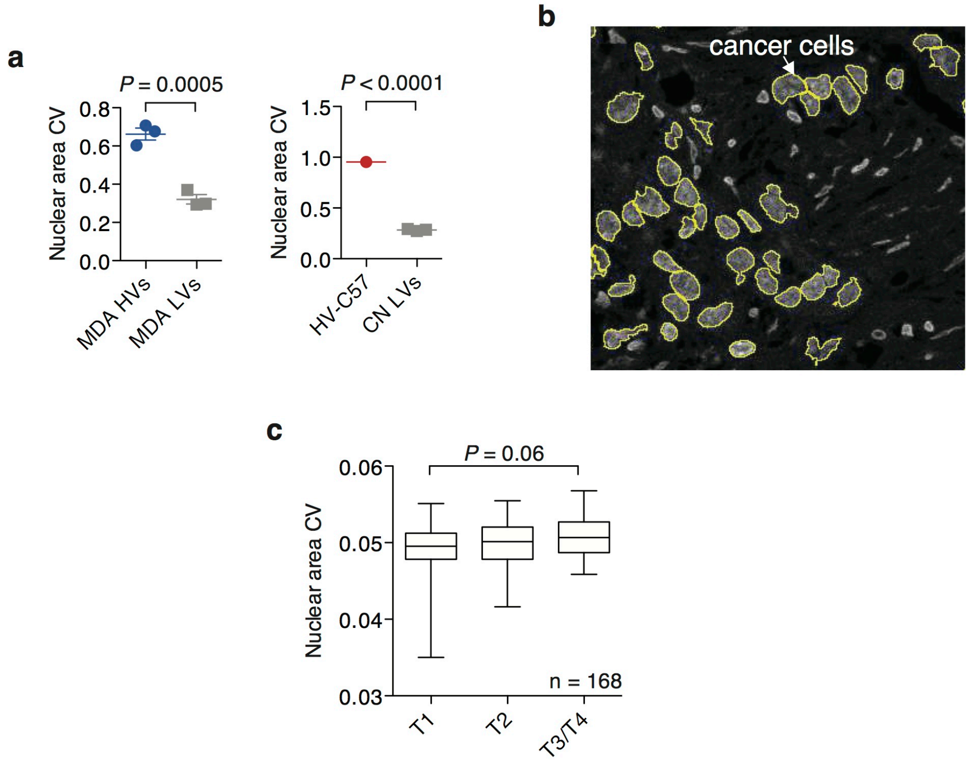

Supplementary Figure 3. Nuclear area coefficient of variation in human breast cancer

a, Nuclear area variability as measured by automated image analysis. Line indicates median. *P*-values were derived using two-sided *t*-test. **b**, Example of tumor core nuclei gating from NCI CDP Breast Cancer Progression Tissue Microarray that excludes stromal cells. **c**, NCI CDP Breast Cancer Progression Tissue Microarray was stained with DAPI, imaged by confocal microscopy, and analyzed for nuclear area coefficient of variation for each breast cancer core. *P*-value was derived using one-sided *t*-test. Median, interquartile range, minimum, and maximum are depicted by box plots.

Supplementary Figure 4

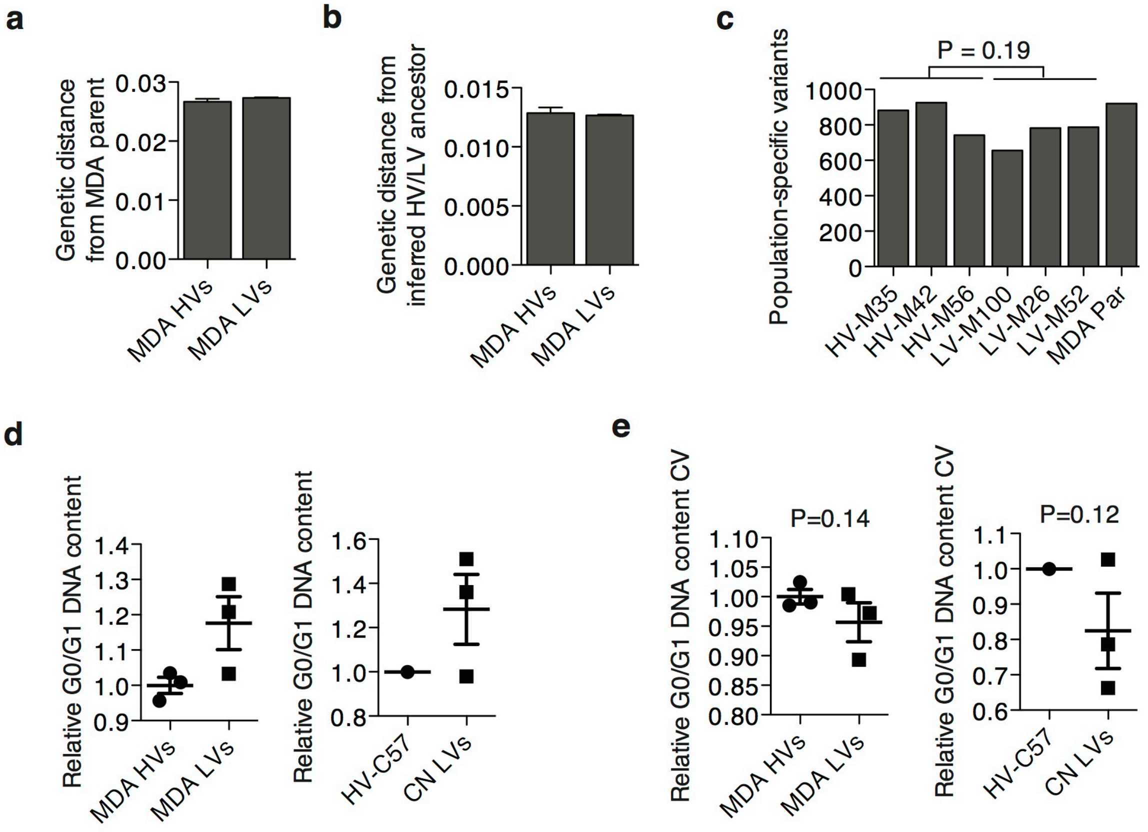

Supplementary Figure 4. Characterization of genomic alterations in highly variable subpopulations

a, **b**, Genetic distance of populations from MDA parent (**a**) or from inferred HV or LV common ancestor (**b**) as determined by Nei's genetic distance. **c**, Variants found only in the indicated populations at any frequency greater than zero were counted. **d**, **e**, Fixed cells were stained with DAPI and analyzed by flow cytometry to measure DNA content. G_0/G_1 peak was analyzed for mean population DNA content (**d**) and variability in DNA content (**e**). P-values were derived using one-sided t-test. Error bars indicate s.e.m. unless otherwise indicated.

Supplementary Figure 5

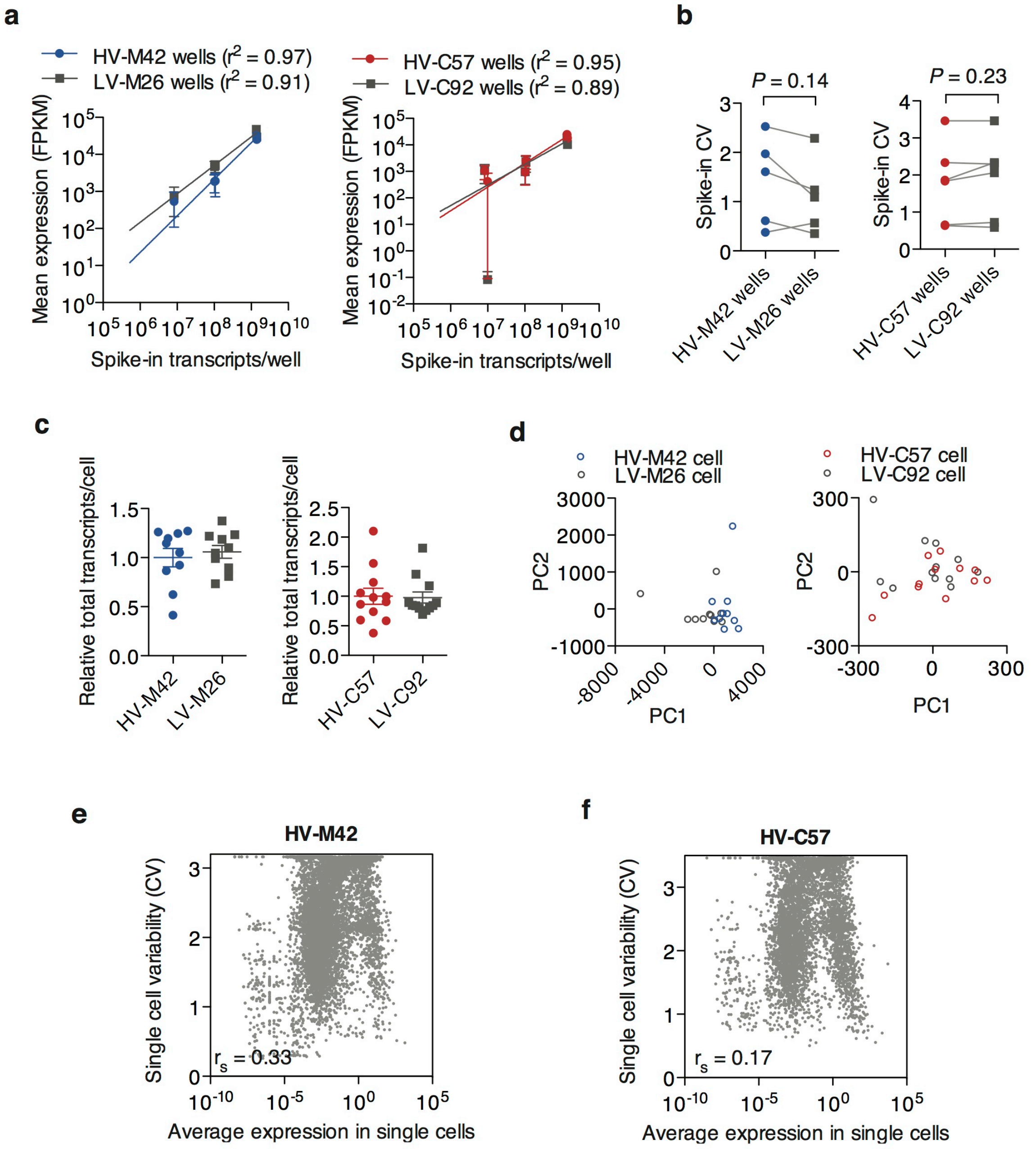

Supplementary Figure 5. Single-cell RNA-sequencing of clonal subpopulations

a, Mean expression of spike-in transcripts per well as assessed by single cell sequencing was plotted against known spike-in concentrations. **b**, Coefficient of variation of spike-in transcripts between wells containing the indicated population of cells was calculated. P-values were derived using two-sided paired *t*-test. **c**, Relative transcript abundance per cell from MDA-derived subpopulations (left) and CN-derived subpopulations (right). **d**, Principal component analysis was performed on single-cell transcript expression profiles from MDA-derived subpopulations (left) and CN-derived subpopulations (right). **e**, **f**, Relationship between average expression in single cells and variability of 8,218 transcripts in 10 HV-M42 cells (**e**) or variability of 5,826 transcripts in 12 HV-C57 cells (**f**). Spearman's correlation is shown. Error bars indicate s.e.m. unless otherwise indicated.

Supplementary Figure 6

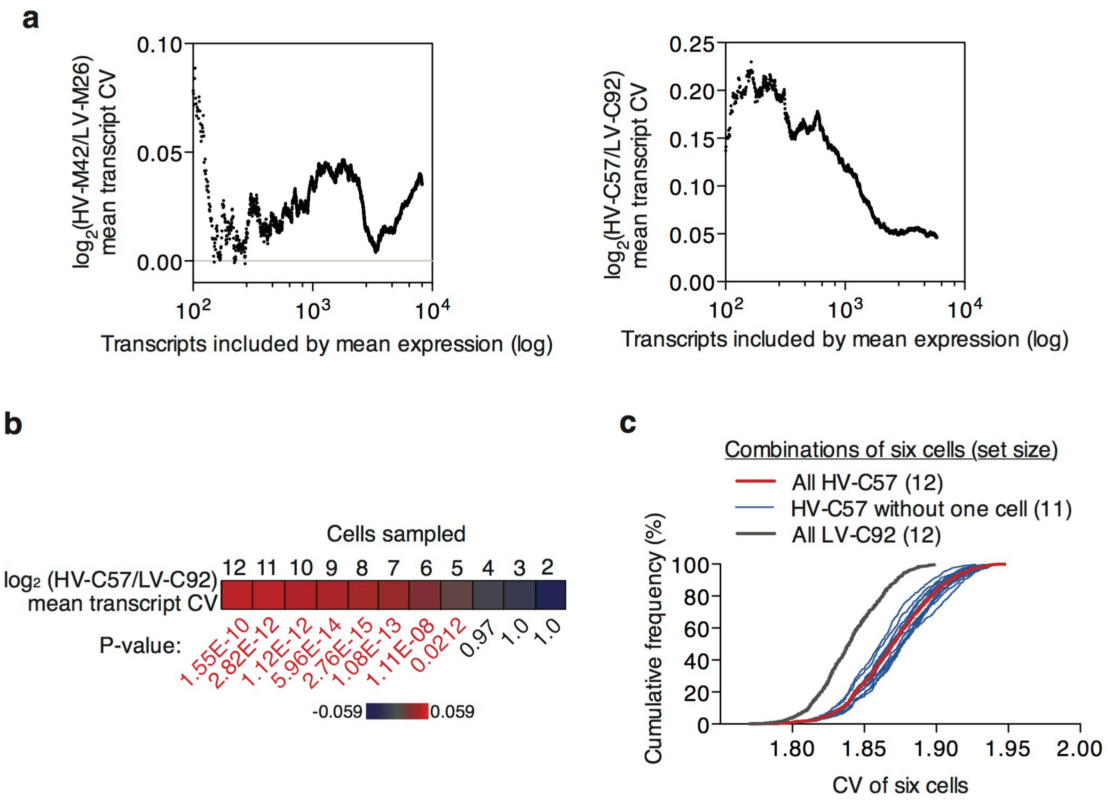

Supplementary Figure 6. Characterization of cell-to-cell transcript expression variability

a, Log-ratios of transcript CV mean were calculated by using varying number of transcripts, ordered by descending mean single cell expression. Analysis was performed using a minimum of 100 transcripts. All cells, 10 for each MDA-derived HV and LV subpopulation (left) and 12 for each CN-derived HV and LV subpopulation (right), were used for analysis. **b**, Log-ratios of transcript CV mean were calculated by sampling MDA-derived (top) and CN-derived (bottom) HV or LV subpopulations 10 times using different set sizes and calculating the mean transcript CV for HV and LV subpopulations. P-values were derived using one-sided paired *t*-test. All transcripts were used for analysis. Significant p-values are in red. Color scales are shown. **c**, All possible combinations of six cells were sampled from the 12 CN-derived HV cells with one specific cell removed (set size of 11), and cumulative frequency distribution of samplings was plotted (blue, 12 possible combinations to exclude each of the 12 cells). Samplings of six cells from all HV cells (red, 1 possible combination to include all cells) or all LV cells (grey, 1 possible combination to include all cells) are shown.

Supplementary Figure 7

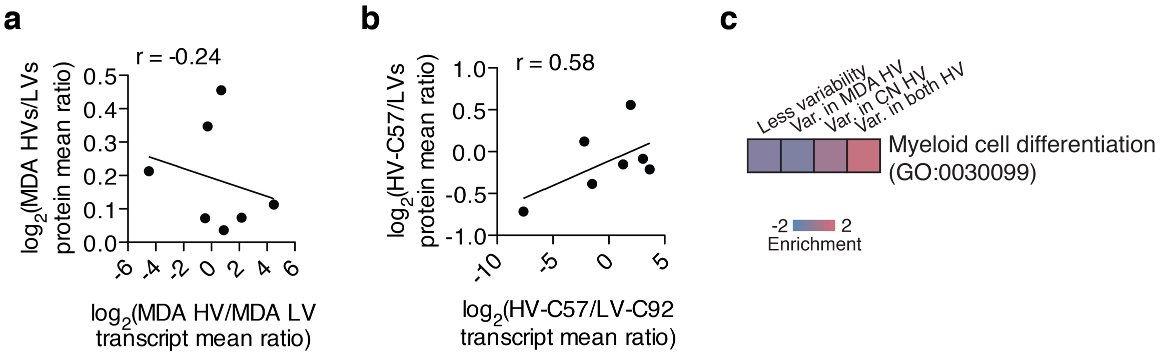

Supplementary Figure 7. Additional analyses of highly variable transcripts

a, **b**, Protein mean expression log-ratios as measured by flow cytometry from MDA-derived (**a**) or CN-derived (**b**) was compared to transcript mean expression log-ratios as measured by single cell RNA sequencing. Pearson's correlation coefficient is shown. **c**, Pathway analysis was used to identify gene sets that are enriched in transcripts with high expression variability in either or both HV subpopulations. Enrichment scale is shown.

Supplementary Figure 8

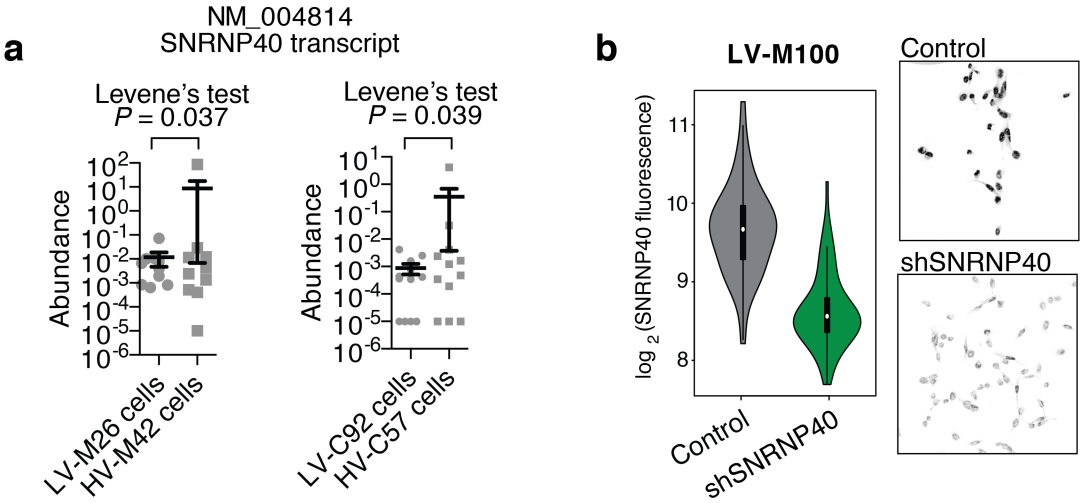

Supplementary Figure 8. Increased SNRNP40 transcript and protein expression coefficient of variation

a, Single cell expression of SNRNP40 transcript (NM_004814). P-value to test variation was performed using Levene's test. Error bars indicate s.e.m. **b**, LV-M100 cells were depleted of SNRNP40 by shRNA-mediated knockdown and were quantitated for SNRNP40 protein expression by immunofluorescence confocal microscopy. Representative gray scale fluorescence images are shown.

Supplementary Figure 9

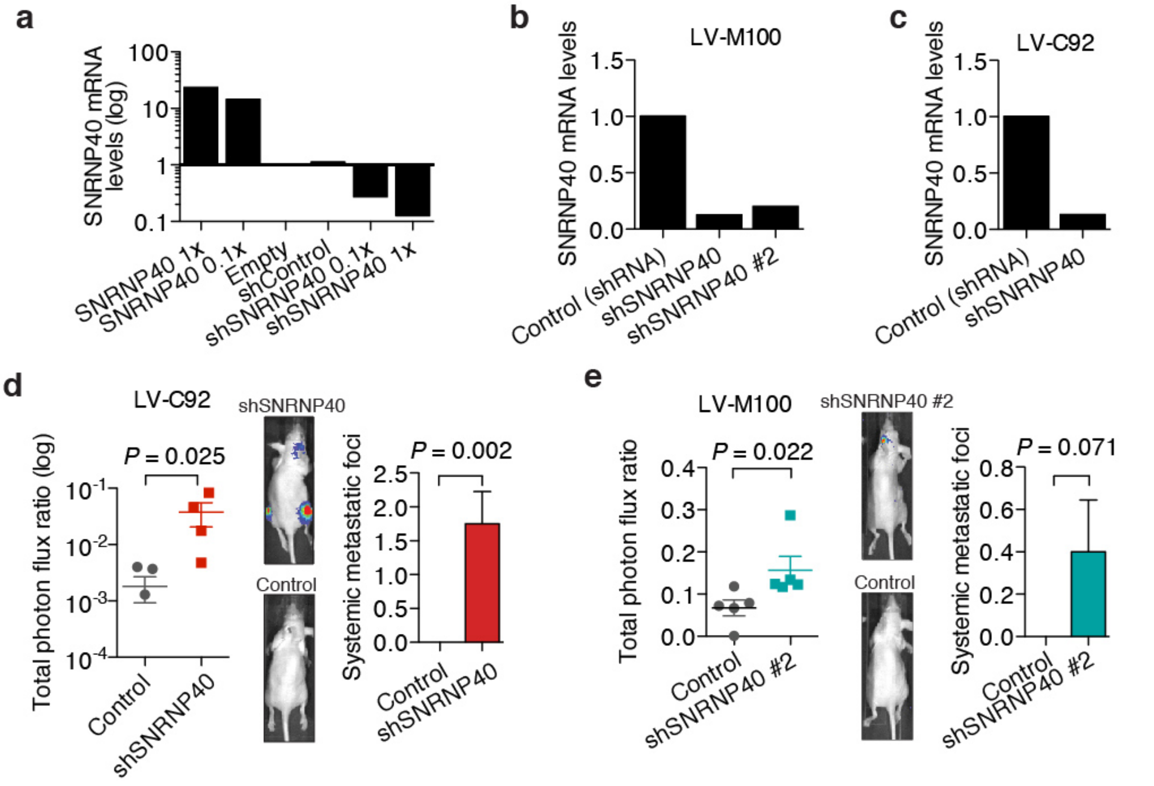

Supplementary Figure 9. Increased SNRNP40 transcript and protein expression coefficient of variation

a, Populations of LV-M100 cells were generated with over-expression of SNRNP40, shRNA-mediated knockdown of SNRNP40, empty vector, or control shRNA vector at varying titers to generate various population-level expression of SNRNP40 as measured by qRT-PCR. **b**, **c**, shRNA-mediated knockdown was quantitated by qRT-PCR in LV-M100 cells (**b**) and LV-C92 cells (**c**). **d**, **e**, 2×10^5 LV-C92 cells (**d**) and with SNRNP40 depletion were inoculated by intracardiac injection and monitored by bioluminescence at day 40 for LV-C92 and day 56 for LV-M100. Representative mouse bioluminescence is shown. Systemic metastatic foci per mice were counted. P-values were derived from one-sided t-test.

Supplementary Figure 10

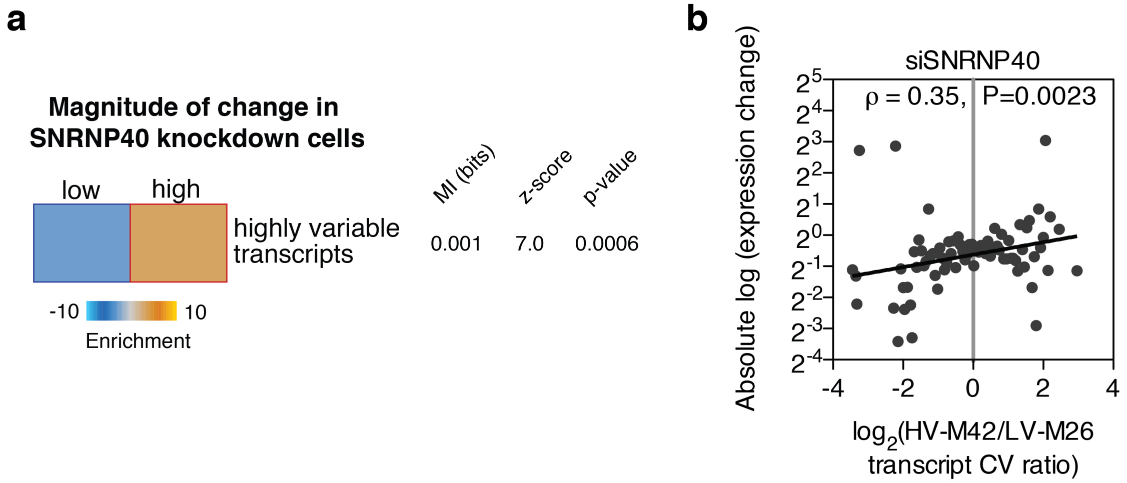

Supplementary Figure 10. SNRNP40-dependent gene expression correlates with gene expression variability seen in highly variable subpopulations.

LV-M100 cells were transfected with two siRNAs targeting SNRNP40 and two control siRNAs and processed for gene expression analysis. **a,** Absolute log-fold change was calculated to determine the magnitude of change. High magnitude change was defined as absolute log-fold change > 1. Highly variable transcripts were defined using single-cell RNA-sequencing experiments as $\log_2$(HV-M42/LV-M26 transcript CV ratio) > 1.5. The calculated mutual information value (in bits) and z-score are provided. P-value was derived using two-sided Fisher's exact test. Also shown are the enrichment scores, presented as logP (positive for enrichments and negative for depletions), where P is calculated from hypergeometric distribution (shown as a heatmap with blue and gold showing depletion and enrichment, respectively). **b**, Gene expression variability ratios of 6,906 transcripts derived from single-cell RNA-sequencing were binned into 100 bins. For transcripts in each bin, the absolute log-fold expression change of SNRNP40 siRNA treatment relative to control siRNAs was calculated, averaged, and plotted. Robust linear

regression is shown in black. Two-sided spearman’s correlation and corresponding p-value are shown.

Supplementary Table 1

RefSeq	Gene	MDA HV/LV log2-ratio	CN HV/LV log2-ratio
NM_020464	NHSL1	2.885	1.965
NM_015542	UPF2	1.460	1.993
NM_005837	POP7	2.141	1.085
NM_000967	RPL3	2.838	0.310
NM_145796	POGZ	3.074	0.058
NM_032307	C9orf64	1.812	1.265
NM_006990	WASF2	1.920	1.083
NM_199287	CCDC137	1.461	1.531
NM_001031804	MAF	1.416	1.537
NM_001242312	FAM124A	1.757	1.190
NM_001127175	MRO	1.876	1.054
NM_001077203	SENP7	1.921	0.980
NR_033702	MYNN	2.874	0.002
NM_173510	CCDC117	1.796	1.014
NM_022098	XPNPEP3	1.326	1.473
NM_001198530	TCF7L2	1.444	1.336
NM_139135	ARID1A	1.821	0.956
NM_021033	RAP2A	2.126	0.642
NM_021130	PPIA	1.176	1.576
NM_003111	SP3	1.182	1.569

Supplementary Table 1. Top 20 highly variable transcripts by single cell RNA-sequencing. The top 20 most highly variable transcripts were determined by ordering the average of the $\log_2$ ratios from the two parental lines. Genes validated at the protein level by flow cytometry are highlighted yellow.

Supplementary Table 2

RefSeq	Gene	MDA HV/LV log2-ratio	CN HV/LV log2-ratio	IPA Network connecting spliceosomal nodes
NM_015484	SYF2	1.212	1.456	2
NM_003092	SNRPB2	1.252	1.100	2
NM_004814	SNRNP40	0.745	1.250	6
NM_003016	SRSF2	1.067	0.378	3
NM_004941	DHX8	0.459	0.807	0

Supplementary Table 2. Top 5 highly variable spliceosomal transcripts by single cell RNA-sequencing. The top 5 most highly variable spliceosomal gene transcripts were determined by ordering the average of the log_2 ratios from the two parental lines. Ingenuity Pathway Analysis (IPA) network connecting spliceosome nodes indicates the number of highly variable spliceosomal genes that had been described to directly interact with the indicated gene. SNRNP40 is highlighted in yellow.
